# The Influence of Cerebral Arterial Circle on Prominent Hypointense Vessel Signs in Patients With Internal Carotid Artery Occlusion

**DOI:** 10.3389/fneur.2021.753877

**Published:** 2022-01-14

**Authors:** Lei Yang, Ling Yu, Wei Qin, Yue Li, Shuna Yang, Xuanting Li, Wenli Hu

**Affiliations:** ^1^Department of Neurology, Beijing Chao-Yang Hospital, Capital Medical University, Beijing, China; ^2^Department of Endocrinology, Beijing Chao-Yang Hospital, Capital Medical University, Beijing, China

**Keywords:** susceptibility-weighted imaging (SWI), carotid artery occlusion, collateral circulation, stroke, circle of Willis

## Abstract

**Background and Purpose:**

Previous studies on the presence of asymmetrical prominent cortical and medullary vessel signs (APCV/APMV) and collateral circulation in patients with internal carotid artery occlusion internal carotid artery occlusion (ICAO) are rare, and the conclusions are inconsistent. Our study aimed to investigate the relationship between the presence of APCV/APMV and collateral circulation in patients with ICAO.

**Methods:**

Patients with acute ischemic stroke with ICAO were recruited in this study. All 74 patients were divided into two groups depending on the presence of APCV and APMV. The status of the cerebral arterial circle (CAC) was graded as poor or good. The poor CAC was defined as MCA was invisible. Severe stroke was defined as cerebral watershed infarction (CWI) or territorial infarction (TI). Clinical and radiological markers were compared between these two groups. Logistic regression was used to investigate the association between the APCV/APMV and clinical and radiological markers.

**Results:**

A total of 74 patients with ICAO were enrolled. Forty-three patients (58.1%) presented with an APCV and APMV was found in 35 (47.2%) patients. Compared with patients with non-APCV, patients with APCV had a more severe stroke (*P* = 0.038) and had a significantly higher incidence of poor CAC (*P* = 0.022) than those with APCV. Patients with APMV had a more severe stroke (*P* = 0.001). Logistic regression showed that poor CAC was independently associated with APCV and severe stroke were independently associated with APMV.

**Conclusions:**

Our study demonstrates that poor CAC was independently associated with the presence of the APCV in patients with ICAO. Severe stroke was independently associated with the APMV.

## Introduction

The nature of cerebral hemodynamics in patients with internal carotid artery occlusion (ICAO) depends mainly on the cerebral arterial circle (CAC) or circle of Willis, which determines the long-term prognosis of the patients. The prevalence of acute internal carotid artery occlusion in acute ischemic stroke is reported to be 6–15% (1). Acute ischemic stroke with ICAO is usually caused by cerebral hypoperfusion or embolism, while hypoperfusion is responsible for chronic ICAO (2).

Collaterals determine initial clinical presentation and outcomes in both acute and chronic patients with ICAO. The primary collaterals include the arterial segments of the CAC, anterior communicating artery, and posterior-communicating artery. The secondary collaterals consist of the ophthalmic arteries and leptomeningeal vessels (3). MCA flow positivity is an important sign of robust collateral circulation in patients with ICAO. Therefore, it is important to evaluate the area of hypoperfusion and collateral circulation in patients with ICAO.

Susceptibility-weighted imaging (SWI) is a useful tool in the assessment of acute ischemic stroke. It is highly sensitive to paramagnetic substances such as deoxyhemoglobin and has been applied to evaluate brain tissue at risk of infarction (4). In the area of hypoperfusion, there is an increase in the oxygen extraction fraction (OEF, deoxyhemoglobin/oxyhemoglobin ratio) within tissue capillaries and cerebral veins. This intravascular deoxygenation leads to a signal drop along the course of cerebral veins, which contributes to the prominent hypointensity of draining veins on SWI.

In the recent studies with SWI, the prominent hypointense vessel signs (PVS), including asymmetrically prominent cortical and medullary veins (APCV and APMV), were described in patients with acute ischemic stroke. APCV has been hypothesized to represent the ischemic penumbra and correlate well with perfusion parameters of raised mean transit time and time to peak (5–7). Moreover, APCV usually disappears after effective cerebral blood reperfusion (8). Studies on APMV are relatively rare and results show that APMV was associated with the presence of arterial occlusion, larger infarct volume (9), and stroke severity (10, 11).

Up to now, there have been some controversies about the presence of PVS with radiological factors and prognosis. Some studies suggested that the presence of PVS is associated with better collateral circulation, lower initial NIHSS scores, and good prognosis (12–14). However, most cases have shown a negative correlation between PVS and good prognosis in ischemic stroke (5, 7, 10, 11, 15–17). There are also other studies that reported that no correlation was found between the presence of APCV and prognosis (9, 18). Another systematic review reported that the prominent vessel signs were not a predictive factor for recanalization after reperfusion therapy for acute ischemic stroke (19).

In most studies, the PVS is present in patients with severe intracranial arterial stenosis or occlusion. It indicates that collateral circulation plays an important role in the presence of PVS and outcome in patients with ICAO. However, previous studies on the PVS and collateral circulation in acute stroke patients are rare, especially in patients with ICAO. Park et al. suggested that the presence of APCV is associated with better collateral circulation (14), while Verma et al. suggested that the APCV correlates with poor-leptomeningeal collateralization (17).

At present, the significance of the PVS in patients with ICAO is unclear. Therefore, we aimed to investigate the relationship between the PVS and the primary collateral in acute ischemic stroke patients with ICAO.

## Methods

### Study Population

We recruited consecutive patients who were diagnosed with ischemic stroke or transient ischemic attack (TIA) with internal carotid artery occlusion at the Department of Neurology in Beijing Chao-Yang Hospital, Capital Medical University, from January 2018 to December 2019. Patients included in the study met the following criteria: (1) admitted within 3 days after stroke onset; (2) unilateral internal carotid artery occlusion was confirmed by computed tomography angiography (CTA); (3) all the patients received MRI, DWI, SWI, CTA, and time-of-flight MR angiography (MRA); (4) SWI and DWI performed between 24 h and 3 days after admission.

The exclusion criteria included: (1) contralateral internal carotid artery or middle cerebral artery stenosis (more than 50% diameter loss) or occlusion; (2) SWI sequence in the MRI protocol showed poor quality; (3) patients with a definite cardioembolic source (e.g., atrial fibrillation, recent myocardial infarction, dilated cardiomyopathy); (4) patients received intravenous or intra-arterial thrombolytic therapy.

### Ethics Statement

The design of this study was approved by the Ethics Committee of Beijing Chao-Yang Hospital, Capital Medical University. Since this study was retrospective, informed consent of the included patients was not required.

### Demographic and Clinical Assessments

Demographic features and risk factors were recorded: hypertension (defined as receiving medication for hypertension or a blood pressure >140/90 mmHg on repeated measurements), diabetes mellitus (DM, defined as receiving medication for DM or diagnosed at discharge), current smoker, alcohol use, history of stroke, and history of coronary heart disease (CAD). National Institutes of Health Stroke Scale (NIHSS) score was measured at the time of admission and discharge.

All patients underwent laboratory testing: urea nitrogen (BUN), creatinine (Cr), uric acid (Ur), glycosylated hemoglobin (HbA1c), low-density lipoprotein cholesterol (LDL), high-density lipoprotein cholesterol (HDL), triglycerides (TG), and homocysteine (HCY). All patients received a cardiac evaluation, including electrocardiogram and heart ultrasound.

### Magnetic Resonance Imaging Protocol and Assessment

Brain MRI scans were performed within 3 days after admission, including SWI, DWI, fluid-attenuated inversion recovery (FLAIR), T1-weighted imaging (TI), T2-weighted imaging (T2), and MRA. Brain MRI was performed with a 3.0 T imager (Siemens, Erlangen, Germany) and a 3.0 T imager (GE, discovery 750, America).

Susceptibility-weighted imaging (Siemens) data were collected with a gradient echo sequence using the following parameters: section thickness 1.2 mm with no interslice gap, time repetition/time echo = 28/20 ms, flip angle = 15°, field-of-view = 230 × 230 mm, and voxel size = 1.2 × 1.2 × 1.2 mm. The parameters of SWI (GE) were as follows: section thickness 2.6 mm with 1.2 mm interslice gap, time repetition/time echo = 29.6/20.1 ms, flip angle = 15°, field-of-view = 230 × 230 mm, and voxel size = 1.2 × 1.2 × 1.2 mm.

Two investigators (Yue Li, Shuna Yang), who were blinded to clinical data, independently reviewed the MRI sequences including DWI, SWI, WMH, and CTA. The disagreement was resolved by a third investigator (Wenli Hu). SWI was assessed before DWI to avoid a reading bias.

### Assessments of Cerebral Arterial Circle/Collateral Circulation

The primary collaterals include the arterial segments of the CAC, anterior communicating artery, and posterior communicating artery. As a primary collateral circulation, CAC was the main object of our study.

**Figure 1:**
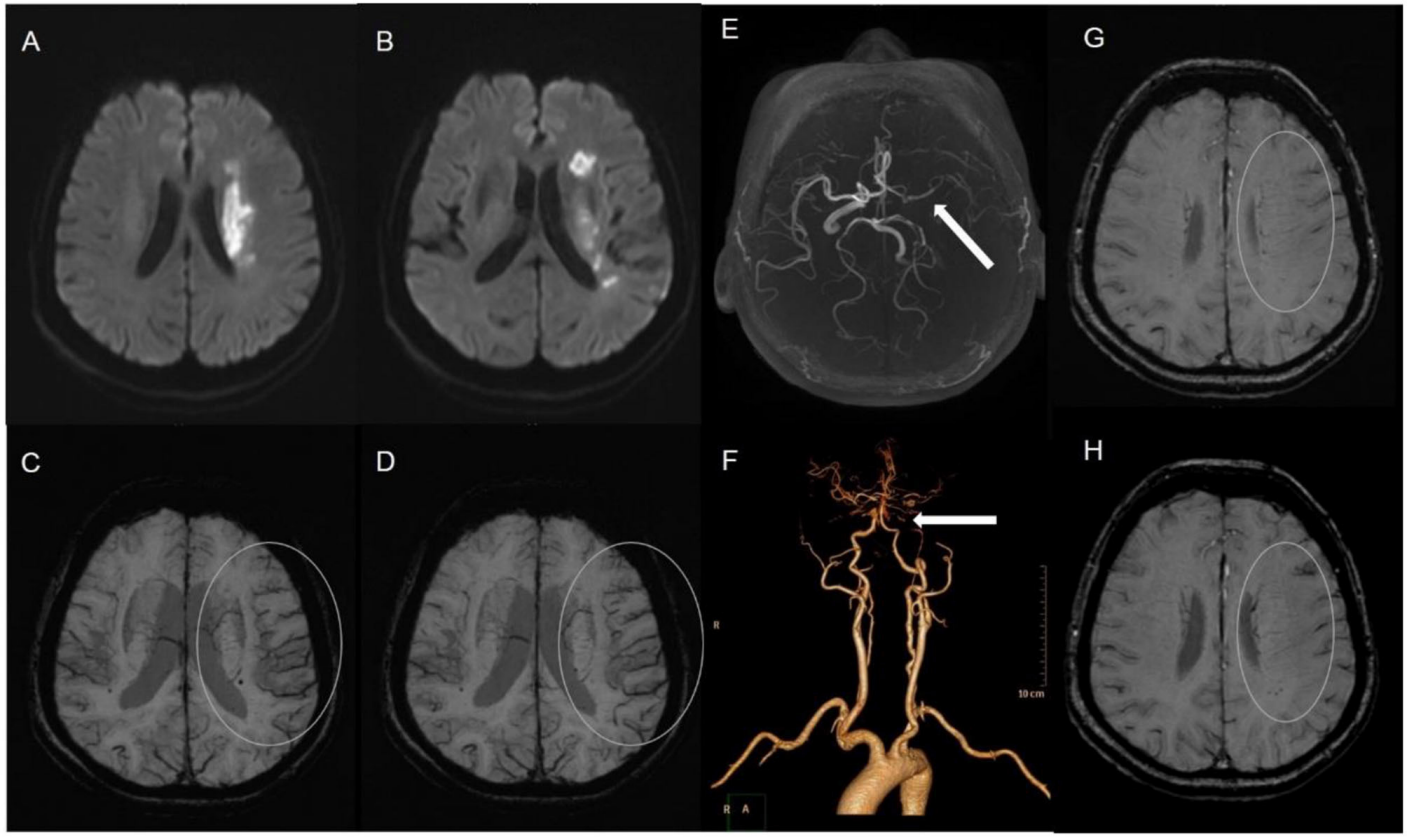
A 62-female patient with left CWI **(A,B)**. SWI indicates significantly more and larger vessels with a greater signal loss (APCV) in the left hemisphere [**(C,D)** white circle]. CTA showed left internal carotid artery occlusion [**(F)** white arrow]. The status of cerebral arterial circle was graded as good with the presence of contrast filling from the M1 to M3 of MCA and anterior cerebral artery [**(E)** white arrow]. The SWI showed prominent medullary veins hypointense signals (APMV) in the left hemisphere [**(G,H)** white circle].

**Figure 2:**
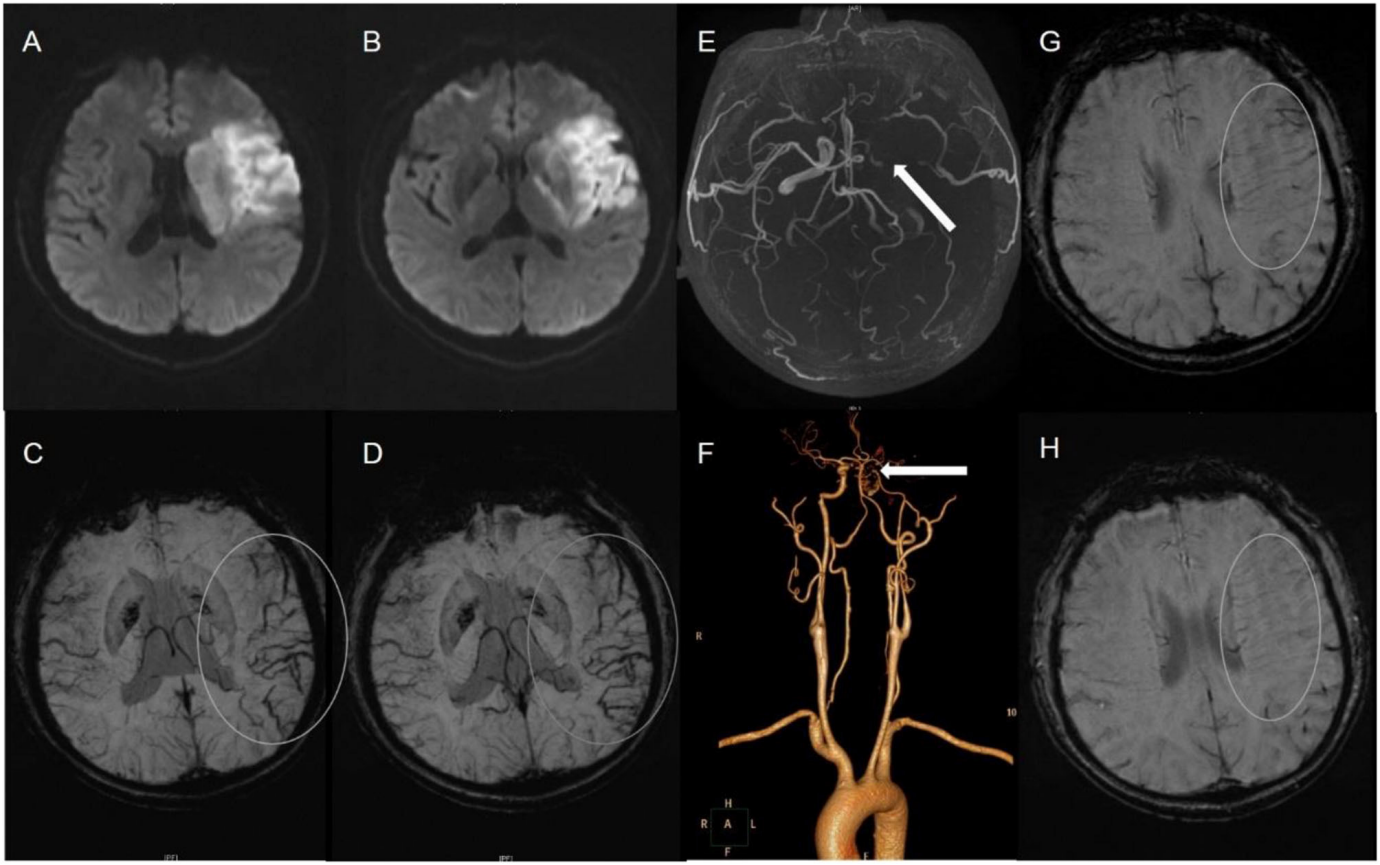
A 59-male patient with left TI **(A,B)**. SWI indicates APCV in the left hemisphere [**(C,D)** white circle]. CTA showed left internal carotid artery occlusion [**(F)** white arrow]. The status of cerebral arterial circle was graded as poor with MCA was invisible [**(E)** white arrow]. The SWI showed prominent medullary veins hypointense signals (APMV) in the left hemisphere [**(G,H)** white circle].

**Figure 3:**
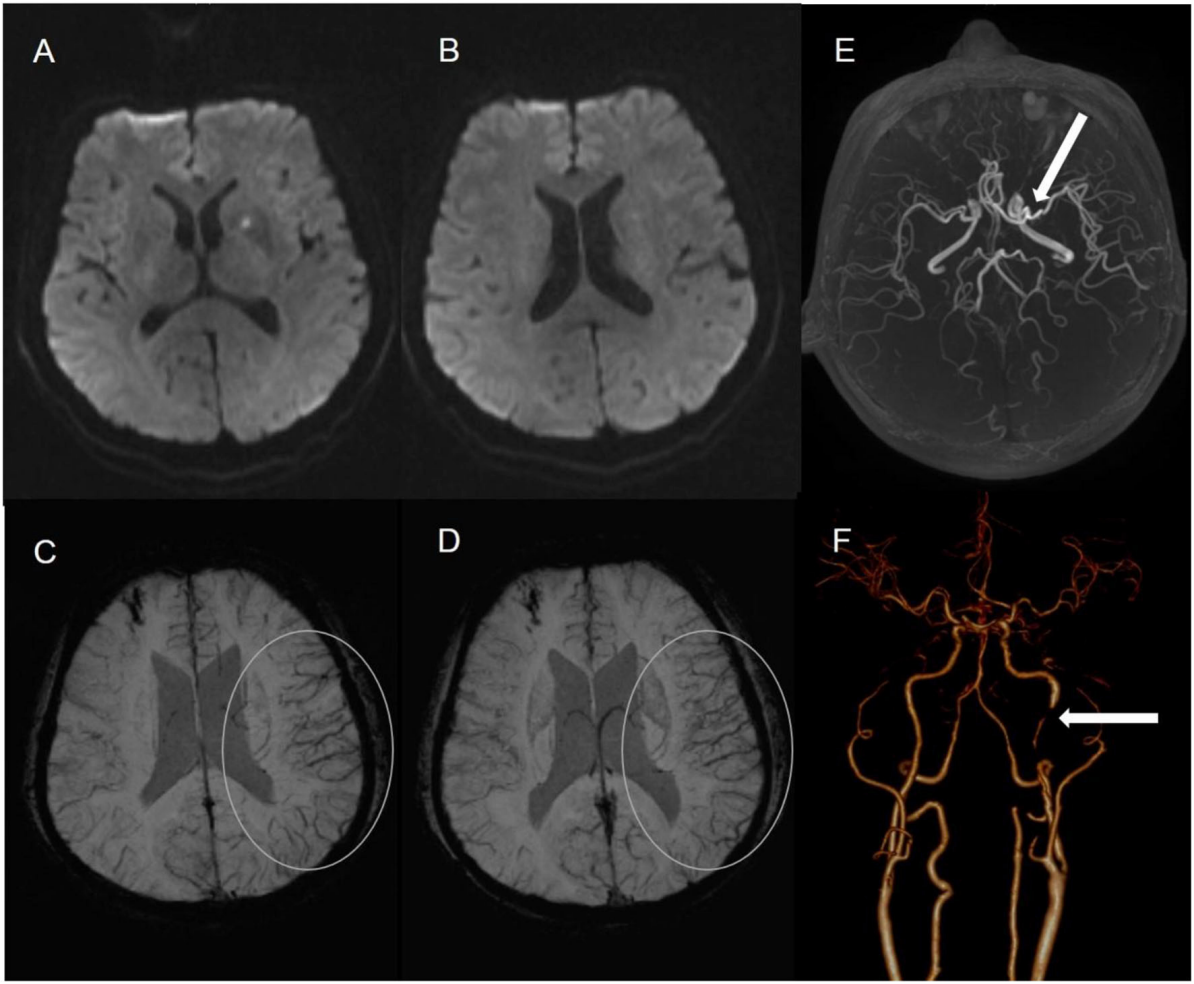
A 61-male patient with left LI **(A,B)**. SWI indicates APCV in the left hemisphere [**(C,D)** white circle]. CTA showed left internal carotid artery occlusion [**(F)** white arrow]. The status of cerebral arterial circle was graded as good with the presence of contrast filling from the M1 to M3 of MCA and anterior cerebral artery [**(E)** white arrow].

The status of the CAC was graded as poor (**Figure 2**) or good (Figures 1, **3**). The poor CAC degree was defined as MCA being invisible on MRA. The good degree was defined as the presence of the M1 to M3 of MCA and anterior cerebral artery (ACA).

### Assessments of PVS

Asymmetrical prominent cortical vessels (17) was defined as more and/or larger vessels with greater signal loss than those in the opposite hemisphere on minimum intensity projection of SWI (Figures 1–3), whereas the same status that occurred along the course of the medullary veins in the deep white matter has been called the APMV (11) (Figures 1, 2).

### Assessments of WMH

Periventricular white matter hyperintensities (PVH) and deep white matter hyperintensities (DWMH) of contralateral were separately assessed using the Fazekas scale (20). Patients were divided into two WMH burden groups by their Fazekas scores as follows: “mild group,” with a PVH/DWMH Fazekas score of 0, 1; “moderate-severe group”, with an PVH/DWMH Fazekas score of 2, 3.

### Assessments of Cerebral Infarction Type

Acute ischemic infarction was defined as lacunar infarction (LI) in the lenticulostriate artery, cerebral watershed infarction (CWI), or territorial infarction (TI) with increased signal on DWI sequence. CWI (21), ischemic lesions between two non-anastomosing main arterial territories, can be classified as either cortical watershed infarction (WSI) or internal WI (IWI). TIA was defined as a transient episode of neurological dysfunction caused by focal brain, without increased signal on DWI sequence.

Imaging data were divided into four grades according to infarct area, grade 0 (TIA), grade 1 (LI), grade 2 (WSI), and grade 3 (TI).

Moreover, according to the imaging finding on DWI, patients with CWI or TI were defined as severe stroke, while TIA and LI were mild stroke.

### Statistical Analysis

Continuous variables with normal distributions were presented as means with SD and compared using the independent-samples *t*-test. Variables with non-normal distributions were presented as medians with interquartile ranges and compared using the Mann-Whitney *U*-test. Categorical variables were compared using the chi-squared test.

Logistic regression analysis was also used to determine independent predictors for APCV and APMV, with vascular risk factors (found significant by the chi-squared test) and clinical data (found significant by the independent samples *t*-test and Mann-Whitney U test) in the model. Statistical analyses were performed SPSS version 20.0 (SPSS Inc., Chicago, IL, USA). *P* < 0.05 was considered statistically significant.

## Results

### Characteristics of the Patients

During the study period, 93 patients with ICAO were identified. After excluding 19 patients due to poor-quality SWI, 74 patients (65.5 ± 10.3 years; range 37–88 years; 75.7% male) were enrolled. Risk factors were determined for the population of the study: 51 patients (68.9%) had hypertension, 36 (48.6%) patients had diabetes, 24 patients (32.4%) had stroke history, 13 (17.6%) had CAD history, 41 (55.4%) were current smokers, and 26 (35.1%) reported alcohol use.

A total of forty-three patients (58.1%) presented with an APCV on SWI and APMV was found in 35 (47.2%) patients. Among the 74 patients with ICAO, 7 (9.5%) had LI, 22 (29.7%) had CWI, and 24 (32.4%) had TI. The remaining 21 patients were diagnosed with TIA. A total of nineteen (25.7%) patients had moderate-severe PVH and 11 (14.9%) had moderate-severe DWMH.

Details of clinical and demographic features of all patients were presented in Table 1.

**Table 1:** Baseline characteristics of enrolled patients.

**Clinical features**	**All patients (*n* = 74)**
Age (years)	65.5 ± 10.3
Male	56 (75.7%)
Hypertension	51 (68.9%)
Diabetes	36 (48.6%)
History of CAD	13 (17.6%)
History of stroke	24 (32.4%)
Current smoker	41 (55.4%)
Alcohol use	26 (35.1%)
NIHSS	2 (1-4)
APCV	43 (58.1%)
APMV	35 (47.2%)
Good CAC	46 (62.2%)
Acute infarction	53 (71.6%)
LI	7 (9.5%)
CWI	22 (29.7%)
TI	24 (32.4%)
SBP (mmHg)	150.9 ± 20.3
DBP (mmHg)	81 ± 13.3
TG (mmol/L)	1.38 (1.07–1.87)
LDL (mmol/L)	2.90 ± 0.89
HbA1c (%)	7.14 ± 1.71
HCY (μmol/L)	15.5 (12–19)
Cr (μmol/L)	68.45 (60.9–75.5)
Ur (μmol/L)	326.9 ± 95.5
PVH (Fazekas) ≧2, *n* (%)	19 (25.7%)
DWMH (Fazekas) ≧2, *n* (%)	11 (14.9%)
MRI (days)	4 (4–6)

*Data are presented as mean ± SD, median (interquartile range) or counts (%). CAD, coronary artery atherosclerosis disease; APCV, asymmetrically prominent cortical veins; APMV, asymmetrically prominent medullary veins; LI, lacunar infarction; CWI, cerebral watershed infarction; TI, territorial infarction; CAC, cerebral arterial circle; TG, triglycerides; LDL, low-density lipoprotein; HbA1c, glycosylated hemoglobin; Cr, creatinine; Ur, uric acid; HCY, homocysteine; PVH, periventricular white matter hyperintensities; DWMH, deep white matter hyperintensities*.

### Characteristics of Patients According to the Presence of APCV and APMV

Clinical and radiologic markers of the two groups according to the presence of APCV and APMV were presented in Table 2.

**Table 2:** Characteristics of patients with ICAO according to the presence of APCV and APMV.

**Clinical features**	**APCV**		***P***	**APMV**		***P***
	**(31, negative)**	**(43, positive)**		**(39, negative)**	**(35, positive)**	
Age (years)	65.7 ± 11.5	65.4 ± 9.5	0.888	65.1 ± 10.5	66.0 ± 10.2	0.702
Male	26 (83.9%)	30 (68.9%)	0.163	31 (79.5%)	25 (71.4%)	0.420
Hypertension	20 (64.5%)	31 (72.1%)	0.487	27 (69.2%)	24 (68.6%)	0.951
Diabetes	12 (38.7%)	24 (55.8%)	0.146	21 (53.8%)	15 (42.9%)	0.345
History of CAD	7 (22.6%)	6 (14%)	0.336	6 (15.4%)	7 (20%)	0.602
History of stroke	13 (41.9%)	11 (25.6%)	0.138	15 (38.5%)	9 (25.7%)	0.242
Current smoker	16 (51.6%)	125 (58.1%)	0.577	23 (59%)	18 (51.4%)	0.514
Alcohol use	10 (32.3%)	16 (37.2%)	0.660	13 (33.3%)	13 (37.1%)	0.732
NIHSS at admission	2 (1–3)	2 (1–5)	0.205	2 (1–4)	2 (1–5)	0.447
Severe stroke, *n* (%)	15 (48.4%)	31 (72.1%)	0.038*	17 (43.6%)	29 (82.9%)	0.001*
Good CAC	24 (77.4%)	22 (51.2%)	0.022	24 (61.5%)	22 (62.9%)	0.907
PVH (Fazekas) ≧2	7 (22.6%)	12 (27.9%)	0.605	10 (25.6%)	9 (25.7%)	0.994
DWML (Fazekas) ≧2	5 (16.1%)	6 (14%)	0.795	4 (10.3%)	7 (20%)	0.239
SBP (mmHg)	149.6 ± 18.4	151.8 ± 21.7	0.465	151.1 ± 19.2	150.7 ± 21.7	0.930
DBP (mmHg)	80.5 ± 11.2	81.4 ± 14.7	0.767	79.2 ± 11.8	83.0 ± 14.7	0.222
TG (mmol/L)	1.6 ± 1.1	1.7 ± 1.2	0.868	1.6 ± 1.0	1.7 ± 1.3	0.617
LDL (mmol/L)	2.74 ± 0.86	3.01 ± 0.91	0.203	2.76 ± 0.91	3.05 ± 0.85	0.168
HbA1c (%)	6.7 ± 1.2	7.46 ± 1.96	0.147	7.08 ± 1.49	7.2 ± 1.96	0.782
HCY (μmol/L)	15.5 (12.3–19)	14.5 (13–19.3)	0.700	16 (13–19.5)	13 (11–17.5)	0.084
Cr (μmol/L)	70.1 (62.5–79.2)	67.1 (59.3–73.6)	0.104	70 (62.2–78.6)	67.1 (59.8–74.2)	0.238
Ur (μmol/L)	79.8 ± 28.5	68.3 ± 15.1	0.372	336 (280–415)	273 (240–341)	0.011

Data are presented as mean ± SD, median (interquartile range) or counts (%). APCV, asymmetrically prominent cortical veins; APMV, asymmetrically prominent medullary veins; CAD, coronary artery atherosclerosis disease; severe stroke, including CWI and TI; CAC, cerebral arterial circle; TG, triglycerides; LDL, low-density lipoprotein; HbA1c, glycosylated hemoglobin; Cr, creatinine; Ur, uric acid; HCY, homocysteine; PVH, periventricular white matter hyperintensities; DWMH, deep white matter hyperintensities. *P < 0.05.

Compared to patients with non-APCV, patients with APCV had a more severe stroke (*P* = 0.038). Patients with non-APCV had a significantly higher incidence of good collateral circulation (*P* = 0.022) than those with APCV. There were no significant differences in terms of age, gender, and vascular risk factors between the APCV and non-APCV groups.

Compared to patients with non-APMV, patients with APMV had a more severe stroke (*P* = 0.001). The level of uric acid was significantly higher in patients without APMV. There was no significant difference between the two groups regarding good collateral circulation. Similarly, there were no significant differences in terms of age, gender, and vascular risk factors between the two APMV groups.

### Association Between APCV and Clinical Data and Radiologic Markers

Multivariable logistic regression analyses showed that, after adjusting for age, history of stroke, and infarction type, the poor CAC was independently associated with APCV. These statistical results are displayed in Table 3.

**Table 3:** Multivariable models (Enter) of the association between APCV and clinical and radiologic markers in patients with ICAO.

**Clinical features**	**OR**	**95% CI**	***P***
Age	1.012	0.962–1.064	0.654
History of stroke	0.604	0.196–1.186	0.380
good CAC	0.270	0.081–0.899	0.033^*^
Grade0-TIA			0.522
Grade1-LI	1.084	0.176–6.675	0.930
Grade2-CWI	2.769	0.661–11.596	0.163
Grade3-TI	1.565	0.411–5.964	0.512

*OR, odds ratio; CI, confidence interval; CAC, cerebral arterial circle; TIA, transient ischemic attacks; LI, lacunar infarction; CWI, cerebral watershed infarction; TI, territorial infarction*.

* *P < 0.05*.

### Association Between APMV and Clinical Data and Radiologic Markers

After adjusting for age, history of stroke, and CAC, logistic regression analyses showed that watershed infarction and territorial infarction were independently associated with APMV. The cerebral arterial circle was not associated with APMV. These statistical results are displayed in Table 4.

**Table 4:** Multivariable models (Enter) of the association between APMV and clinical and radiological markers in patients with ICAO.

**Clinical features**	**OR**	**95% CI**	***P***
Age	1.015	0.963–1.070	0.568
History of stroke	0.947	0.274–3.267	0.931
good CAC	1.897	0.543–6.634	0.316
Grade0-TIA			0.009^*^
Grade1-LI	4.363	0.595–32.006	0.147
Grade2-CWI	9.076	1.769–46.573	0.008^*^
Grade3-TI	16.460	3.127–86.649	0.001^*^

*OR, odds ratio; CI, confidence interval; CAC, cerebral arterial circle; TIA, transient ischemic attacks; LI, lacunar infarction; CWI, cerebral watershed infarction; TI, territorial infarction*.

* *P < 0.05*.

### Combined Analysis of APCV and APMV

Data regarding the combined analysis of APCV and APMV were shown in Table 5. Among the 74 patients, 26 (35.1%) showed concurrent presence of APCV and APMV. There was a significant difference between the four subgroups regarding the proportion of severe stroke (*p* = 0.004). Patients with the concurrent present of APCV and APMV had the highest rate of severe stroke.

**Table 5:** Combined analysis of APCV and APMV.

**Clinical features**	**APCV(+) APMV (–)** ***n* = 17**	**APCV(+) APMV(+)** ***n* = 26**	**APMV(+) APCV(–)** ***n* = 9**	**APMV(–) APCV(–)** ***n* = 22**	***P***
Age (years)	66.2 ± 9.9	64.9 ± 9.4	69.3 ± 12.3	64.3 ± 11.1	0.640
Male	11 (64.7%)	19 (73.1%)	6 (66.7%)	20 (90.9%)	0.224
Hypertension	12 (70.6%)	19 (73.1%)	5 (55.6%)	15 (68.2%)	0.804
Diabetes	12 (70.6%)	12 (46.2%)	3 (33.3%)	9 (40.9%)	0.194
History of CAD	2 (11.8%)	4 (15.4%)	3 (33.3%)	4 (18.2%)	0.566
History of stroke	6 (35.3%)	5 (19.2%)	4 (44.4%)	9 (40.9%)	0.328
NIHSS at admission	2 (1–4)	2 (1–5)	2 (0–8)	2 (1–3)	0.561
Severe Stroke, *n* (%)	8 (47.1%)	23 (88.5%)	6 (66.7%)	9 (40.9%)	0.004^*^
good CAC	7 (41.2%)	15 (57.7%)	7 (77.8%)	17 (77.3%)	0.091

*Data are presented as mean ± SD, median (interquartile range), or counts (%). APCV, asymmetrically prominent cortical veins; APMV, asymmetrically prominent medullary veins; CAD, coronary artery atherosclerosis disease; severe stroke, including CWI and TI; CAC, cerebral arterial circle*.

* *P < 0.05*.

## Discussion

In this study, we found that poor CAC was independently associated with the presence of the APCV in patients with ICAO. Severe stroke was independently associated with the presence of APMV. To our knowledge, this is the first study mainly focused on patients with ICAO and found poor CAC was independently associated with the presence of the APCV.

Patients with ICAO exhibit have different clinical manifestations, ranging from asymptomatic to TIA, and even severe stroke syndromes. The clinical presentation of ICAO patients depends on various factors, especially the availability of collateral circulation. The robustness of the primary circle of Willis determines initial clinical presentation and resultant outcomes in patients with ICAO. SWI is a useful tool in assessing cerebrovascular disease and does not require the application of contrast agents. SWI provides a rough estimate of tissue hypoperfusion with the hypointense cortical veins sign in the ischemic territory due to increased concentration of deoxyhemoglobin. After vascular occlusion, the contradiction between oxygen supply and demand in the hypoperfused region may lead to an elevated oxygen extraction fraction and subsequent increased level of deoxyhemoglobin in the vessel.

The level of vein oxygenation and the presence of APCV/APMV in the ischemic territory may be influenced by collateral circulation (4). If collateral circulation is good enough and oxygen supply is sufficient, APCV can disappear. A study including patients with hyperacute ischemic stroke reported that APCV disappeared after full recanalization (8). Our study is consistent with a study including patients with 33 ischemic stroke with M1-segment occlusion of the middle cerebral artery, which reported that the presence of extensive APCV correlates with poor-leptomeningeal collateralization (17).

However, Park et al. insisted that the presence of APCV is associated with better collateral circulation (14). This research included patients with the internal carotid artery or middle cerebral artery M1 occlusion. The moderate degree of collateral flow was defined as the presence of contrast filling from the cortical branch of M3 to the M2 branch within the Sylvian fissure. The good degree was defined as the presence of contrast filling from the distal branch of M3 to M1.

However, anterograde flow *via* primary collaterals (CAC) is important in patients with ICAO (22), whereas the retrograde flow of secondary leptomeningeal collaterals is more important in patients with MCA occlusion. Different collateral circulation evaluation methods may be the reason for the different results.

The relationship between the presence of APMV and stroke severity and outcome in patients with ischemic stroke is still not clear. Payabvash et al. (9) found that APMV was more strongly associated with larger infarct volumes. Yu et al. (10) reported that the ACVS and AMVS were correlated to the infarct size and the presence of AMVS was independently related to the stroke severity and poor outcome. Mucke et al. (11) found that the APMV on SWI was associated with increased initial stroke severity and Wang et al. (23) also reported APMV was significantly higher in the poor outcome group.

Similar to the aforementioned researches that our study found imaging biomarkers of severe stroke, watershed infarction, and territorial infarction, correlated with the presence of APMV in patients with ICAO. Although we did not study the relationship between the prominent vessel signs and outcome, previous studies suggest that patients with large infarction had a poor prognosis. In contrast, Kim and Langheinrich (12) reported both ipsilateral and contralateral thalamostriate vein susceptibilities showed a strong inverse correlation with presenting NIHSS score in patients with hyperacute stroke. The inconsistency of results may be related to the time between the onset of symptoms and SWI examination. An animal study (24) demonstrated that both cerebral blood flow (CBF) and OEF varied greatly over time with no consistent difference in values between penumbral and the eventually infarcted tissues. A case provided by An et al. showed that a patient who presented with aphasia and mild right hemiparesis was received an SWI scan at 5, 48 h, and 12 weeks after symptom onset (25). The prominent veins signs were visible immediately adjacent to the ischemic lesion at 5 h, while the contrast between the veins and surrounding tissues was reduced at 48 h and completely disappeared at 12 weeks. Deep medullary veins originate in the subcortical white matter and mainly drain the blood from the deep white matter vein flows to the subependymal medullary veins. The presence of AMVS on SWI occurs in the patients with IACO reflected poor collateralization. Deep white matter is the most easily involved site of hypoperfusion, which corresponds to the common site of anterior circulation watershed infarction. More studies will be needed to determine the evolution of the prominent vessel signs overtime on SWI and its clinical significance.

Currently, asymmetric prominent vessel signs have been hypothesized to represent the ischemic hypoperfusion area. Whether the hypoperfusion area can be saved may be related to the time of cerebral ischemia. After the ischemic event, the ischemic territory demonstrates increased oxygen extraction fraction and manifests as APCV/APMV. In the hyperacute phase, the presence of APCV suggests a good prognosis, and APCV can disappear after recanalization treatment (6, 8, 12, 26). After that, the persistence of prominent vessel signs might indicate that ischemic tissue cannot be saved and the prognosis is poor (5, 11, 16, 17). The evolution process of the prominent vessel signs overtime on SWI has not been determined, and the window period for the existence and disappearance of the prominent vessel signs is also not clear. In the future, prospective studies of different time groups may shed some light on the puzzle.

There are several limitations to our study. First, this is a cross-sectional single-center and small sample study which prevents us from making a causal inference. Second, we did not include patients in the hyperacute phase who required thrombolysis and thrombectomy, so we did not observe the imaging changes on SWI before and after treatment. Third, APCV/APMV assessment was performed using a visual assessment less precise than quantitative evaluation and we did not calculate the volume, so we choose the current method as an alternative. Fourth, we did not perform perfusion imaging which was a good indicator for cerebral tissue perfusion. Last, we did not follow up on patients and did not investigate long-term outcomes.

## Conclusions

In conclusion, we found that poor CAC was independently associated with the presence of the APCV in patients with ICAO. Severe stroke was independently associated with the presence of APMV.

## Data Availability Statement

The raw data supporting the conclusions of this article will be made available by the authors, without undue reservation.

## Ethics Statement

The studies involving human participants were reviewed and approved by the Ethics Committee of Beijing Chao-Yang Hospital, Capital Medical University. Written informed consent for participation was not required for this study in accordance with the national legislation and the institutional requirements.

## Author Contributions

LYa planned the study, collected data, and wrote the manuscript. LYu and WQ collected the data and revised the manuscript. YL, SY, and XL analyzed the data and revised the manuscript. LYa and WH interpreted the data and revised the manuscript. WH designed the study and revised the manuscript. All authors read and approved the final manuscript.

## Conflict of Interest

The authors declare that the research was conducted in the absence of any commercial or financial relationships that could be construed as a potential conflict of interest.

## Publisher's Note

All claims expressed in this article are solely those of the authors and do not necessarily represent those of their affiliated organizations, or those of the publisher, the editors and the reviewers. Any product that may be evaluated in this article, or claim that may be made by its manufacturer, is not guaranteed or endorsed by the publisher.
